# Insulin-Like Growth Factor Replacement Therapy for Diabetic Neuropathy: Experimental Basis

**DOI:** 10.1155/EDR.2003.257

**Published:** 2003

**Authors:** Douglas N. Ishii, Sean B. Lupien

**Affiliations:** 1 Department of Biomedical Sciences and Department of Biochemistry and Molecular Biology Colorado State University Fort Collins Colorado 80523 USA


*Experimental Diab. Res.*, 4:257–269, 2003
Copyright © Taylor and Francis Inc.
ISSN: 1543-8600 print / 1543-8619 online
DOI: 10.1080/15438600390249709

### Insulin-Like Growth Factor Replacement Therapy for Diabetic Neuropathy: Experimental Basis

**Douglas N. Ishii and Sean B. Lupien**

*Department of Biomedical Sciences and Department of Biochemistry and Molecular Biology, Colorado State University, Fort Collins, Colorado, USA*

### INTRODUCTION

Diabetic neurological complications continue to progress in a substantial fraction of patients despite best efforts at glycemic control. The development of adjuvant treatments to supplement diet, exercise, oral hypoglycemic agents, and insulin is urgently needed and may do much to enhance the quality of patient life. The neurobiology of insulin-like growth factors (IGFs) has been studied in animals, and a loss of IGF activity produces neurological disorders that mimic the disturbances of diabetic neuropathy. The theory that a decline in IGF neurotrophic activity is pathogenic for diabetic neuropathy has efficiently generated many testable hypotheses. The theory predicts and tests show that IGF levels are reduced in diabetic primates, including humans, and that IGF gene expression is reduced throughout the peripheral and central nervous system in diabetic rodents. Tests further show that replacement doses of IGFs can prevent an array of diabetic neurological disturbances in the peripheral and central nervous system. These data point to a common etiology for central and peripheral neurological disturbances. It is of considerable practical and theoretical interest that IGF treatment is effective independently of ongoing hyperglycemia and metabolic imbalance. These observations are in line with emerging clinical data showing that new drugs can be developed specifically for the treatment of diabetic complications, albeit not targeted at glycemic control. The pharmacokinetic and safety profiles support the movement of IGFs toward clinical trials.

Received 10 December 2002; accepted 3 February 2003.

This work was supported by the National Institute of Diabetes and Digestive and Kidney Disease grant R01 DK53922 and Centers for Disease Control and Prevention grant R49/CCR811509. SBL was supported in part by a fellowship from the Colorado Institute for Research in Biotechnology.

Address all correspondence to Dr. Douglas N. Ishii, Department of Biomedical Sciences, Colorado State University, Fort Collins, CO 80523, USA. E-mail: dnishii@aurogen.com

### CLINICAL DIABETIC NEUROPATHY

There are two types of diabetic patients. Type II diabetes is previously known as non–insulin-dependent diabetes mellitus (NIDDM) or adult-onset diabetes. Generally striking late in adulthood, these patients produce normal or elevated amounts of insulin, but are resistant to insulin. Type II diabetes has a 5% to 7% incidence in the general population, and comprises more than 90% of all diabetic patients, or about 14 million people in the United States alone. The incidence is rising as a consequence of the increasing population age, industrialization, and obesity. Furthermore, Hispanics and African-Americans have a higher overall incidence than Caucasians, and their proportion in the general population is increasing. The remaining 10% have type I diabetes, previously known as insulin-dependent diabetes mellitus (IDDM) or juvenile-onset diabetes. They are unable to produce sufficient amounts of insulin due to pancreatic insufficiency, in most cases believed to arise as an autoimmune disorder.

Type I and type II diabetic patients are at risk for diabetic neuropathy. The major pathological feature is a dying-back axonopathy that involves both unmyelinated and myelinated axons. There is a dwindling of axon calibers, reduced axonal transport, loss of synapses, loss of axons, and, ultimately, loss of neurons. Daily wear-and-tear on the nervous system requires ability for nerve regeneration, and the poor nerve regeneration associated with diabetes may explain the loss of synapses, dying-back axonopathy, and loss of neurons. There is reduced conduction velocity in about 90% of patients, but its relationship to clinically meaningful neuropathy remains uncertain because a significantly smaller fraction of patients progress to diabetic neuropathy, and axons can regenerate even when conduction is blocked by tetrodotoxin. The sensory, sympathetic, and motor systems may be variably afflicted.

Muscular atrophy and weakness can develop secondary to motor involvement. Autonomic neuropathy includes gastroparesis with abdominal bloating and pain, loss of bladder tone that sometimes requires catheterization, abnormal cardiovascular function, silent myocardial infarct, and sudden death. Half of affected males have impotence. The most prevalent form of this complication is a symmetrical sensory neuropathy. Burning unremitting pain, such that a patient cannot bear even the weight of a bed sheet, is sometimes encountered. With progression, there is loss of sensory function, leading to the inability to do simple tasks, such as turn the pages of a book. Inability to perceive pain and touch as well as impaired proprioceptors results in an increased tendency for injuries. In conjunction with poor wound healing and increased susceptibility to gangrene, approximately 100,000 limb amputations are performed on diabetic patients each year (Bild et al., 1989; Litzelman et al., 1993). For other manifestations of clinical neuropathy, reviews and monographs on diabetic neuropathy are available (Thomas and Tomlinson, 1993; Vinik et al., 1996; American Diabetes Association, 1993).

### INTENSIVE GLUCOSE THERAPY DOES NOT PREVENT THE PROGRESSION OF DIABETIC COMPLICATIONS IN A LARGE FRACTION OF PATIENTS

The Diabetes Control and Complications Trial (DCCT) has shown that intensive insulin therapy reduces the incidence of neuropathy in 60% of highly selected type I diabetic patients (DCCT Research Group, 1993). This is a very important clinical finding. Unfortunately, neuropathy continues to progress in 40% of patients, despite the best current method for diabetic treatment, showing that there is a need for new treatments in addition to glycemic control. A treatment that could ameliorate, prevent, and/or reverse diabetic neuropathy would be a major therapeutic advance, and would help reduce the nearly $100 billion overall cost of health care for diabetic patients. A disproportionately high fraction of this amount is expended for those patients with complications such as neuropathy.

The development of supplemental treatments for diabetic complications is particularly desirable, because intensive insulin therapy is not benign, and is associated with a threefold increased risk of life-threatening hypoglycemia. Extreme caution is warranted because 43% of severe hypoglycemic episodes are found to occur during sleep. Diabetic patients with neuropathy may be particularly susceptible to severe hypoglycemia under intensive insulin therapy (Hoeldtke et al., 1982; Santiago et al., 1984). These patients may have impaired autonomic function and, thereby, a diminished counterregulatory system to oppose hypoglycemia. Mortality can be increased (Teutsch et al., 1984).

Oral monotherapies have been unable to achieve the goal of intensive therapy in type II diabetic patients, but a combination of insulin, oral agents, and diet can partially reduce diabetic complications (UK Prospective Diabetes Study Group, 1998). Intensive therapy reduced hemoglobin (Hb)$A_{1C}$ levels by approximately 1% below results achieved with conventional therapy, resulting in a 35% reduction in the risk of complications. Therefore, complications continue to progress in a large fraction of patients in spite of the best current methods of glucose control. The incidence of major hypoglycemic adverse events is 2.3% of patients per year. Thus, it is very important for both type I and type II diabetic patients to manage blood glucose as close to normal as possible, and therapies to supplement glycemic control to specifically treat diabetic complications are much needed.

### SUPPLEMENTAL THERAPIES THAT DO NOT TARGET GLYCEMIC CONTROL ARE CLINICALLY EFFECTIVE IN PREVENTING DIABETIC COMPLICATIONS

High blood pressure and microalbuminuria are risk factors for the progression of nephropathy in diabetic patients. Like other diabetic complications, it has long been believed that nephropathy is due mainly to hyperglycemia. However, recent data challenge this belief. There is a 50% reduction in end-stage renal disease and mortality in patients with type I diabetes treated with angiotensin-converting enzyme inhibitors (Lewis et al., 1993). Moreover, there is a 70% reduction in the progression to nephropathy in type II diabetic patients with microalbuminuria when treated with angiotensin-II receptor blockers (Parving et al., 2001). Control of hypertension is important as well. The IGF data are consistent with these observations that diabetic complications can be prevented independently of glycemic state.

### OVERVIEW OF THE IGF SYSTEM

The IGF genes are quite large and each is unique in vertebrates. Alternative exon usage, splicing, and multiple poly(A) termination sites produce multiple IGF-I and IGF-II transcripts. Because all IGF transcripts encode the complete prepro-IGF molecule, it is believed that the multiple transcripts facilitate complex, tissue-selective regulation of gene expression. The IGF-I gene is under growth-hormone control in certain tissues such as liver, but is also responsive to developmental signals, nutritional status, diabetes, aging, and neural activity. The IGF-II gene is likewise responsive to developmental signals, nutritional status, diabetes, aging, and neural activity. However, it is not responsive to growth hormone. In the adult rat, the highest levels of IGF-II gene expression are found in brain, spinal cord, and peripheral nerves. It is expressed in liver in humans, but not rats, due to absence of the hepatic promoter. Hence, circulating IGF-II is abundant in humans but essentially absent in adult rodents. The IGF-I gene is likewise expressed in adult brain, spinal cord, peripheral nerves, and liver, but its expression is more widespread among other tissues as well. IGF-II is by far the predominant IGF in brain.

IGF-I and IGF-II are protein hormones (molecular weight 7.5 kDa) comprised of a single polypeptide chain held together by three intrachain disulfide bonds. IGFs are members of the insulin gene family; they are endocrine, autocrine, and paracrine factors. Both IGF-I and IGF-II bind to the type I IGF receptor, and it is believed that most actions of IGFs are through this receptor. The type I receptor is a plasma membrane–bound heterodimer homologous to the insulin receptor where the extracellular $\alpha$ subunits contain the IGF-binding domain, and an intracellular domain of the $\beta$ subunit is a tyrosine kinase. The type I receptor is present on all or virtually all neurons, and is localized to the cell body, axons, and nerve terminals. This receptor on brain neurons is smaller than those on peripheral tissues due to decreased glycosylation of the $\alpha$ subunits, and does not appear to gate glucose uptake. In addition, IGF-II binds the type II receptor that is comprised of a single polypeptide located in the plasma membrane and devoid of tyrosine kinase activity. It appears to be involved in IGF-II degradation and lysosomal targeting, and may also signal possibly through a G-protein mechanism. Because their three-dimensional structures resemble that of insulin, IGFs can cross-occupy insulin receptors, but this occurs only at supraphysiological concentrations.

There are six members of an IGF-binding protein (IGFBP-1 through -6) family that sequester IGFs. Circulating IGFs form a trimeric complex, predominantly with IGFBP-3, and the acid labile subunit. IGFs in the extracellular fluid, on the other hand, are generally in the form of dimers together with one of the IGFBPs. It is believed that tissue proteases may act on these dimers to regulate the availability of free IGFs.

It is instructive to consider the major additive sources of IGFs for the nervous system. Neurons have access to IGFs produced in brain, spinal cord, and peripheral nerve. In addition to these autocrine/paracrine sources of IGFs, circulating endocrine sources of IGFs, primarily from liver, can provide further support for peripheral neurons. Because the signaling type I IGF receptor binds to both IGF-I and IGF-II, these two neurotrophic ligands form a redundant back-up system for one another. The onset of neuropathy may be slower in humans because hepatic IGF-II production provides back-up neurotrophic support to partially offset the loss of circulating IGF-I in younger diabetic patients, whereas the onset may be more rapid in adult diabetic rats due to the absence of circulating IGF-II. It will be seen that in diabetes there is a progressive loss of autocrine, paracrine, endocrine, and redundant IGF neurotrophic support. Such loss is proposed to increase the risk of neuropathy.

### NEUROBIOLOGY OF IGFs

A brief review of the neurobiology of IGFs helps to explain how IGFs are implicated in diabetic neuropathy. The strength of this model is that diabetic neurological disturbances can be rationally understood from the biochemistry and physiology of IGF action in the nervous system.

IGFs are neurotrophic factors that can support and prevent damage to neurons. The neurotrophic properties of IGF-I and IGF-II were initially discovered in the 1980s. IGF-I and -II were found to induce neurite outgrowth and support survival in cultured sensory, sympathetic, and human neuroblastoma cells (Recio-Pinto and Ishii, 1984; Ishii et al., 1985; Recio-Pinto et al., 1986). These studies were soon extended to show that IGFs can support a wide variety of central nervous system (CNS) as well as peripheral nervous system (PNS) neurons.

The cloning and sequencing of the rat IGF-I (Shimatsu and Rotwein, 1987) and IGF-II (Soares et al., 1985, 1986) genes permitted examination of their tissue-specific and developmental expression. The IGF-II gene is selectively expressed at the highest levels in the brain, spinal cord, and peripheral nerves among tissues of the adult rat. The IGF receptors are found on neurons as well as glial cells. The expression of the IGF-II gene is closely correlated with the development of synapses (Ishii, 1989). IGFs can increase neurite (axon and dendrite) growth in cultured neurons (Recio-Pinto and Ishii, 1984; Recio-Pinto et al., 1986), by increasing the expression of the genes that encode structural proteins of axons, such as tubulins and neurofilaments (Mill et al., 1985; Wang et al., 1992). Highly purified recombinant human IGF-I and IGF-II can increase axon growth and support survival of neurons cultured from various parts of the central (brain and spinal cord) and peripheral nervous systems. Overexpression of IGF-I in brain of transgenic mice results in brains 55% larger than normal (Mathews et al., 1988), and mouse strains with reduced IGF-I levels have underdeveloped brains (Noguchi et al., 1986; Beck et al., 1995). IGFs appear to be able to act on all or virtually all neurons in the body.

Various data suggest that the normal role of IGFs is to help maintain the nervous system in adult mammals, including humans. IGF treatment can help repair damaged nervous systems. Administration of recombinant human IGF-I (Kanje et al., 1989) or IGF-II (Glazner et al., 1993) was found to significantly increase the rate of sensory nerve regeneration in adult rats. IGF-II administration increases motor nerve regeneration as well (Near et al., 1992). IGFs are normally produced in nerves, and IGF genes are turned on to help damaged nerves regenerate (Glazner et al., 1994). Following sciatic nerve transection in neonatal rats, IGF-II administration can prevent loss of motoneurons (Pu et al., 1999a). These results demonstrate that IGF treatment can help repair damaged peripheral nerves in a mammal.

Although the central effects of IGFs are not discussed here, what has emerged is the concept that IGFs are circulating and CSF neurotrophic factors that provide general support to virtually all neurons within the PNS and CNS. Other neurotrophic factors, such as neurotrophins (nerve growth factor [NGF], brain-derived nerve factor [BDNF], neurotropin-3 [NT-3]) provide additional support for select populations of neurons. Review articles may be consulted on the neurobiological actions of IGFs (Recio-Pinto and Ishii, 1988; de Pablo and de la Rosa, 1995; D'Ercole et al., 1996; Ishii and Pu, 1999). These data show the broad potential that IGFs have to treat the many different types of cells of the central and peripheral nervous systems.

### LOSS OF IGF CAUSES NEUROLOGICAL DISTURBANCES THAT MIMIC THOSE OBSERVED IN DIABETES

Blocking of IGF activity in normal, nondiabetic animals will produce neuropathy with characteristics similar to that observed in diabetes. For example, anti-IGF antibodies can block sensory and motor nerve regeneration in normal rats (Kanje et al., 1989; Near et al., 1992; Glazner et al., 1993), and nerve regeneration is impaired in diabetes. Neuron loss may occur in clinical diabetes in both the peripheral and central nervous systems, and this is observed in rats treated with an anti-IGF antiserum (Pu et al., 1999a) and in IGF-I–null mice (Beck et al., 1995). Conduction velocity (rate at which electrical signals travel down nerves) and axonal diameters are reduced in diabetic patients, and also in IGF-I–knockout mice (Rabinovsky et al., 1996; Gao et al., 1999). IGF-I administration can reverse the low motor and sensory nerve conduction velocity in these mice. These data show the loss of IGF activity is a risk factor for neuropathy independently of hyperglycemia.

IGFs can increase $\alpha$-tubulin, $\beta$-tubulin, 68-kDa neurofilament, and 170-kDa neurofilament gene expression (Mill et al., 1985; Wang et al., 1992). Consequently, a decline in IGF gene expression in diabetes is conjectured to cause diabetic biochemical disturbances, including reduced neurofilament and tubulin production in nerves, and tubulins are needed for assembly of microtubules. Neurofilaments regulate axonal diameters, and axonal diameters are reduced in diabetes. The absence of adequate amounts of these major cytoskeletal proteins may result in loss of synapses and axons. Tubulins further provide tracks on which axonal transport depends, and axonal transport is disrupted in diabetes. The metabolic need would be greatest for the longest axons, and this may underlie the length-dependent axonopathy in diabetes.

### THEORY FOR IGF INVOLVEMENT IN THE PATHOGENESIS OF DIABETIC NEUROPATHY

IGFs support the types of neurons afflicted in diabetes, namely sensory, sympathetic, and motor. The theory is proposed that both insulin and IGFs are required to maintain the proper functioning of the nervous system, and that there is a decline in insulin and IGF activity in diabetes that predisposes to neuropathy (Ishii, 1995). In brief, insulin, IGF-I, and IGF-II are proposed to provide redundant neurotrophic support for neurons. This theory predicts that (i) IGF activity is reduced in diabetes; (ii) neuropathy can be prevented by administration of IGFs; (iii) central and peripheral neurological disturbances may share a common etiology involving IGFs; (iv) neuropathy might be treatable irrespective of hyperglycemia; (v) differences in the manner in which rats and humans develop neuropathy can be explained at least in part by a species-specific difference in the pattern of IGF gene expression; and (vi) an age-dependent loss of IGF may explain the age-dependent risk of neuropathy. Multifactorial risk is inherent in this formulation, and the total IGF activity in an individual would depend, for example, on regulation of IGF gene expression, IGF transcript processing, IGF protein synthesis and turnover rates, IGF BP levels, proteases that may govern availability of IGF sequestered to binding proteins, IGF receptors, postreceptor signaling, and other factors. Predictions and tests of the theory are discussed below.

### IGF Levels are Reduced in Animal and Human Diabetes

*Humans and Rhesus Monkeys*

IGF levels are reduced in diabetic patients. Early clinical studies did not control for age, and failed to find a decline in IGF activity in diabetes. However, later studies, using age-matched patient groups, found that IGF-I level is significantly reduced by 40% to 50% in type I as well as type II diabetes (Tan and Baxter, 1986; Arner et al., 1989; Ekman et al., 2000). IGFBP-1 circulating levels are elevated, and this is expected to sequester and reduce the activity of IGFs (Crosby et al., 1992). Diabetic patients with neuropathy have lower serum IGF-I levels versus diabetic patients without neuropathy or nondiabetic patients (Migdalis et al., 1995; Guo et al., 1999). Reduced numbers of IGF-I receptors are found on red blood cells of diabetic patients (Haruta et al., 1989; Migdalis et al., 1995). Serum IGF-I levels are not correlated with glycemic control, measured as $HbA_{1C}$ levels, in type I diabetic patients treated with insulin (Ekman et al., 2000). This observation may explain why neuropathy is not better controlled in insulin-treated patients.

It is critical to recognize that there is an age-dependent decline of IGFs in humans in the later decades of life (Hall and Sara, 1984). This may explain the age-dependence of clinical neuropathy (Pirart, 1978), and the increased incidence of neuropathy after the fourth decade of life. Thus, neurons suffer the loss of IGF neurotrophic activity as a consequence of diabetes, and IGF levels decline further with advancing age slowly over decades.

Neuropathy may be less prevalent in type I juvenile diabetics who are adolescents or young adults, because IGF-I and IGF-II activities remain relatively high in these age groups. However, in ketotic episodes IGF-I levels transiently decline, and there is transient neuropathy (Rieu and Binoux, 1985).

Young rhesus monkeys are lean and have normal glucose tolerance. As animals age, they become obese and develop impaired glucose tolerance, but have normal or slightly elevated insulin levels. Later, they become overtly type II diabetic. It is fascinating that there is a decline in IGF-I activity with every stage in the progression towards diabetes (Bodkin et al., 1991). IGF-I levels are observed to decline in the prediabetic state *prior* to overt hyperglycemia. Thus, IGF-I levels are not correlated with glucose levels in neither monkeys nor humans. Neuropathy is known to develop (Cornblath et al., 1989). The rhesus monkey provides important support for the theory in animals genetically close to humans, and shows further the relationship between age-dependent decline in IGF activity and age-dependent risk of diabetic neuropathy.

*Type I Diabetic Rats*

IGF-I gene expression is profoundly reduced in liver, adrenal glands, and spinal cords of streptozotocin (STZ)-diabetic rats, a model of type I diabetes (Ishii et al., 1994). IGF-II gene expression is reduced in diabetic rat brain (Wuarin et al., 1996). The significant decrease in poly(A)+RNA content per milligram brain tissue (Wuarin et al., 1996) may be related to the progressive cerebral atrophy in the brains of diabetic patients (Araki et al., 1994). IGF-I and IGF-II mRNA content is reduced in sciatic nerves early after the induction of diabetes (Wuarin et al., 1994), most likely in Schwann cells (Pu et al., 1995). IGF-I mRNA and its receptor mRNA levels are reduced in spinal cord, superior cervical ganglia (Bitar et al., 1997; Bitar and Pilcher, 1998), and dorsal root ganglia (Craner et al., 2002). Rats quickly develop neuropathy because of a profound loss of neurotrophic activity involving insulin, IGF-I, and IGF-II. IGF-II activity is developmentally down-regulated (Soares et al., 1985; 1986), insulin activity is reduced experimentally, and IGF-I activity falls secondary to diabetes.

*Type II Diabetic Rats*

The ZDF (fa/fa) rats provide a genetic model of type II diabetes. These rats are obese and become spontaneously hyperinsulinemic and diabetic at about 5 to 6 weeks of age. IGF-II gene expression is reduced in brain, spinal cord, and peripheral nerves in adult diabetic (fa/fa) versus nondiabetic (+/+) littermates (Zhuang et al., 1997; Wuarin et al., 1996). The brains of (fa/fa) rats are smaller. IGF-I gene expression is reduced in liver, but not in spinal cord or nerves. IGF-I, rather than IGF-II, is responsible for regulating the rate of axon elongation during nerve regeneration (Pu et al., 1999). Because nerve IGF-I gene expression is not reduced, nerve regeneration does not appear to be impaired in type II diabetic rats. This is in contrast to the type I diabetic rat where nerve IGF-I gene expression is reduced and regeneration is impaired. These findings in rats is in accordance with clinical data showing that nerve injury is more extensive in type I than type II disease.

These data show that the IGF genes are under complex regulation, and largely independent of hyperglycemia. IGF levels are reduced in the prediabetic state prior to hyperglycemia, and reduced further as a consequence of aging. It is instructive that IGF-I and IGF-II gene expression are increased in cultured hepatocytes directly in response to insulin (Phillips et al., 1991; Goya et al., 2001). Consequently, IGF gene expression may decline in diabetes partially as a consequence of the reduction in insulin activity rather than in response to hyperglycemia. Because IGF-I treatment can normalize brain IGF-II gene expression in diabetic rats independently of hyperglycemia (Armstrong et al., 2000), there may be a cascade in which a reduction of insulin activity leads to a decline in IGF-I and a secondary decline in IGF-II. Because of complex regulation, insulin may be able only to partially restore IGF levels in diabetic patients.

### Replacement IGF Doses Prevent or Reverse Neuropathy in Diabetic Rats

Biochemical and electrophysiological measurements, although interesting, are often difficult to relate directly to symptomatic clinical disturbances. For example, conduction velocity is reduced in virtually all diabetic patients, whereas clinical neuropathy is observed in a much smaller fraction of patients. The relationship of reduced conduction velocity to hyperalgesia or impaired nerve regeneration is unknown. Concerning the latter, nerves are known to regenerate normally even when conduction is blocked with tetrodotoxin. Because the nexus between biochemical and electrophysiology disturbances and symptomatic disturbances are unclear, our studies have focused mainly on functional end points.

*IGF-I and IGF-II Treatments Prevent the Progression of Hyperalgesia*

Supersensitivity to stimuli, or hyperalgesia, is a difficult management problem in diabetic patients. Hyperalgesia is observed in diabetic animals. Mechanical compression elicits paw withdrawal when rats feel uncomfortable, and the threshold force for withdrawal is gradually reduced with the onset of hyperalgesia in STZ-diabetic rats. Osmotic minipumps were implanted under the skin that released either vehicle or solvent in diabetic rats. Infusion of either IGF-I or IGF-II arrested the progression of hyperalgesia (Zhuang et al., 1996). Hyperalgesia also develops in the ZDF (fa/fa) obese and spontaneously diabetic rat. IGF administration can reverse the hyperalgesia in this model of type II diabetes (Zhuang et al., 1997).

*IGF-I and IGF-II Treatments Prevent or Reverse Impaired Nerve Regeneration*

There is daily wear-and-tear on the nervous system, and a need to regenerate nerve terminals. Nerve regeneration is shown to be impaired in diabetes (Longo et al., 1986; Ekstrom and Tomlinson, 1989), and this may directly contribute to the loss of synapses and the dying back of axons.

Because it is known that IGFs normally regulate nerve regeneration (Kanje et al., 1989; Near et al., 1992; Glazner et al., 1993), and that IGF gene expression is reduced in diabetic nerves (Wuarin et al., 1995), the IGF theory (Ishii, 1995) predicts that replacement IGF therapy should prevent impaired nerve regeneration in diabetes. Localized infusion of IGF-I alongside a crush-injured nerve was shown to prevent the impairment of sciatic nerve regeneration in STZ-diabetic rats (Ishii and Lupien, 1995). Additional studies now reveal that systemic infusion of IGF-I or IGF-II can prevent or reverse impaired nerve regeneration (Ishii and Lupien, 1995; Zhuang et al., 1996; 1997). Single daily subcutaneous injections are effective as well. These studies show that systemically administered IGFs can act on nerve cells, and the blood-nerve barrier is not an impediment. Systemically administered IGFs can reach ganglion cells via fenestrated capillaries, have access to nerve terminals, and can probably reach axons at nodes of Ranvier as well.

*IGF-I Treatment Reverses Neuraxonal Dystrophy*

Daily subcutaneous injections of IGF-I can reverse ultrastructural damage to the sympathetic nervous system. This treatment reverses by 86% the neuroaxonal dystrophy in the superior mesenteric ganglion and ileal mesenteric nerves in rats diabetic for 6 months (Schmidt et al., 1999). Neuroaxonal dystrophy is the hallmark of diabetic autonomic neuropathy, characterized by swollen preterminal axons and synapses, and shown in tissues obtained at autopsy from diabetic human subjects (Schmidt et al., 1993).

*IGF-I Treatment Prevents Impaired Wound Healing*

Wounds heal poorly in diabetic patients, often progressing to gangrene and limb amputations. The poor healing is closely associated with development of neuropathy in diabetes. This is not unexpected, because nerve injuries in themselves can produce tissue atrophy, and the health of limbs is dependent on an adequate nerve supply. IGF-I mRNA levels are reduced in the gut mucosa, and gastric lesions heal poorly in diabetic rats (Korolkiewicz et al., 2000). These investigators showed that subcutaneous administration of IGF-I normalizes wound healing in diabetic rats.

*IGF-I Administered Systemically Crosses the Blood-CNS Barrier and Prevents Impaired Learning/Memory*

It is generally regarded that large molecules the size of IGFs (molecular weight 7.5 kDa) do not cross the blood-CNS barrier. It would appear, therefore, that to treat the CNS with IGFs, an access hole would need to be drilled through the skull, a procedure associated with significant risk of surgical mishap or infection. However, recent studies show that IGFs can cross the blood-CNS barrier. In a pioneering study, 8 minutes after injection of $^{125}$I-IGF-I or $^{125}$I-IGF-II into the carotid artery, radioactivity was detected in brain parenchyma by autoradiography of brain slices (Reinhardt and Bondy, 1994). However, the half-life of free IGF is 5 to 10 minutes, and in 8 minutes, approximately half of the radioactive IGF would be metabolized. Because only a small percentage of the radioactivity enters the brain, it was unclear whether the radioactivity detected in brain parenchyma represented radioactive free iodine, iodinated IGF fragments, or intact IGF. This issue was resolved by withdrawing cerebrospinal fluid (CSF) after injecting $^{125}$I-IGF-I into the carotid artery. The radioactivity in the CSF was subjected to sodium dodecyl sulfate (SDS) gel electrophoresis, and some of the radioactivity was observed to migrate together with authentic IGF, showing that intact IGF molecules did cross the blood-CNS barrier (Armstrong et al., 2000). Further study showed that the uptake of IGF into CSF was saturable, indicating that there was an IGF transport molecule at the blood-CNS barrier, and that the uptake process did not require IGF binding to IGFBP nor to the type I IGF receptor (Armstrong et al., 2000; Pulford and Ishii, 2001). Calculations showed that the circulating levels of endogenous IGF could contribute substantially to the IGF in CSF, indicating that there is communication between IGF in blood and brain.

Lesions are present in the central nervous system of diabetic patients. For example, the brain ventricles are enlarged, showing brain shrinkage (Dejgaard et al., 1991; Araki et al., 1994). Learning and memory are impaired in type I (Ryan et al., 1993) and elderly type II (Perlmuter et al., 1984; Tun et al., 1990) diabetic patients. The incidence of dementia is nearly doubled in diabetes, even in patients without cerebrovascular disease (Ott et al., 1999). Like humans, STZ-diabetic rats have reduced brain weight (Lupien et al., 2002), most likely as a consequence of reduced size of the brain transcript pool (Wuarin et al., 1996) and impaired learning/memory (Biessels et al., 1996). IGFs can support hippocampal neurons (Cheng and Mattson, 1992), and the hippocampus plays a role in several forms of learning/memory. IGF is essential for learning/memory, because infusion of an anti-IGF antibody into the lateral ventricles of the brain can block the acquisition of learning/memory (Lupien et al., 2002). IGF gene expression is reduced in the brain and spinal cord in diabetes as discussed above. As in the case of the PNS, these neurobiological observations, based on the IGF theory (Ishii, 1995), led to the prediction that IGF replacement would prevent cognitive disturbances in diabetes. In fact it is found that IGF-I administered by subcutaneous infusion can cross the blood-CNS barrier and prevent impaired learning/memory in diabetic rats (Lupien et al., 2002). IGF treatment may prevent learning/memory disturbances and progression to dementia in diabetic patients.

### IGF Replacement Therapy Prevents Neuropathy During Ongoing Hyperglycemia

Many patients are unable to institute or maintain tight glucose control, and many even with excellent glucose control develop neuropathy. A treatment to prevent diabetic neuropathy that is effective independently of glycemic state would be welcome. Systemic as well as local infusion of IGF-I or IGF-II prevents or reverses hyperalgesia (Zhuang et al., 1996, 1997), impaired nerve regeneration (Ishii and Lupien, 1995; Zhuang et al., 1996), impaired wound healing (Korolkiewicz et al., 2000), and disturbances in learning/memory (Lupien et al., 2002) independently of ongoing hyperglycemia and hypoinsulinemia in type I diabetic rats. In the latter study, rats had ongoing hyperglycemia for 11.5 weeks. A sensitive index of metabolic disturbance is weight loss, and the IGF treatment did not prevent weight loss in these investigations. The doses of IGF in these studies were small physiological replacement doses (5 to 20 $\mu$g/rat/day). Because IGF-I administration can reduce amyloid-beta, which is implicated in the pathogenesis of Alzheimer's disease (Carro et al., 2002), this form of treatment may prevent memory disturbances in Alzheimer's disease as well.

In studies reversing neuraxonal dystrophy, a higher dose of IGF-I was injected, subcutaneously, such that glucose levels were transiently significantly reduced for 1 to 2 hours from 427 mg/dL to 418 mg/dL (Schmidt et al., 1999). A control group was treated with a low dose of insulin to replicate the transient IGF effect on glucose, and insulin, unlike IGF, did not reverse neuraxonal dystrophy. Consequently, neuraxonal dystrophy most likely is specifically reversed by IGF rather than the transient partial reduction in hyperglycemia.

IGF treatment reversed hyperalgesia in the ZDF (fa/fa) model of type II diabetes independently of hyperglycemia, hyperinsulinemia, and weight gain (Zhuang et al., 1997). Therefore, IGF treatment can prevent neuropathy in type I or type II diabetes irrespective of hyperglycemia or direction of weight change. Taken together, the data show that diabetic neurological disorders as well as nephropathy can be treated independently of hyperglycemia. It is clear that extensive biochemical pathways involved in the etiology of neuropathy and nephropathy are not blocked by the consequences of hyperglycemia, and manipulation of growth factor levels can prevent diabetic complications.

### PHARMACOKINETICS OF IGFs

The pharmacokinetics of IGFs in man has been reviewed (Ishii and Pu, 1999). The half-life of IGF-I is 8 to 20 hours due to the formation of complexes with IGFBPs. The daily production is about 40 to 50 $\mu$g per kg per day, and the volume of distribution is about 0.18 L/kg. Clinically efficacious levels can be maintained for at least 16 hours by the subcutaneous route of administration. The pharmacokinetics of IGF-II appears similar from studies in a few patients, but additional study is needed in larger numbers of subjects. These pharmacokinetic parameters indicate that clinical trials may be conducted using a single daily subcutaneous injection of IGFs. Formulation to enhance the stability and extend the duration of IGFs would not be needed.

The daily production rate indicates that the most appropriate IGF dose would be in the range of 15 to 40 $\mu$g/kg/day. This is a replacement dose, and replacement doses are effective in diabetic rats. There are many successful examples of replacement therapy. For example, insulin is replacement therapy for diabetes in type I diabetic patients, glucocorticoids (cortisol, prednisone, dexamethasone) are replacement therapy for Addison's disease, and mineralocorticoids are replacement therapy for adrenal insufficiency.

Many diabetic patients are trained in self-administration of insulin, and could easily adapt to IGF. There is a 50% failure rate within 5 years of initiating use of oral hypoglycemic agents in type II diabetes, and many of these patients convert to insulin use. Thus, 40% of the total diabetic population (types I and II)

self-inject insulin, and daily IGF subcutaneous administration would require a minimum of patient education.

### SAFETY OF IGF ADMINISTRATION

Humans normally have high levels of IGF-I and IGF-II in the circulation. Because the goal of replacement therapy is to return the depleted levels of IGF in diabetic patients back to normal, side effects are not anticipated. The safety and pharmacology of IGFs have been reviewed (Ishii and Pu, 1999). A good deal has been learned about the safety of IGF products. No significant side effects were encountered in diabetic patients treated for 24 weeks with 20 and 40 $\mu$g/kg/day IGF-I (Acerini et al., 1997). Both retinopathy and nephropathy were monitored, and IGF-I did not cause progression. IGF-I is approved for the treatment of Laron dwarfs in Japan. These patients have growth hormone deficiency and an extreme form of insulin resistance; high IGF doses are administered.

Vascular endothelial growth factor (VEGF) is believed to contribute to diabetic proliferative retinopathy, but conclusive evidence of IGF-1 involvement in diabetic retinopathy is lacking. In a careful study, Simo et al. (2002) measured IGF-I as well as VEGF levels in the vitreous fluid from diabetic patients with retinopathy and corrected for increased leakiness of retinal blood vessels. After correcting for serum proteins, it was found that VEGF levels are significantly higher, whereas free active IGF-I levels are lower in the vitreous of patients with proliferative diabetic retinopathy versus nondiabetic subjects. Moreover, IGF-I mRNA levels are lower in diabetic versus nondiabetic retinas in humans (Gerhardinger et al., 2001) and rats (Lowe et al., 1995). Consequently, free IGF-I levels are not correlated with VEGF levels or proliferative retinopathy. These data are consistent with the observation that an increased incidence of retinopathy is not observed in acromegaly patients with or without diabetes (Ballintine et al, 1981; Sonken et al., 1993; Cogessal and Root, 1940). Growth hormone increases IGF-I and GH is increased in acromegaly. In a 2-year prospective study in type II diabetic patients, the level of IGF-I was progressively reduced during insulin treatment, whereas retinopathy progressively worsened; no relationship was established between level of IGF-I and progression of retinopathy (Henricsson et al., 1999). In a 6-year study, Wang et al. (1995) came to the same conclusion. Intraocular IGF-I levels are mainly derived from the circulation because IGF-I as well as serum albumin together leak into the eye in advanced disease (Spranger et al., 2000). Early treatment with an IGF-I analog can prevent early predegenerative changes in diabetic retina (Kummer et al., 2003). A somatostatin analog, ocreotide, was reported to prevent the progression of proliferative diabetic retinopathy (Grant et al., 2000). Gehrs (2001), however, pointed out the problems in the experimental design, including that the baseline retinopathy scores of the control and treated groups were not compared, the treated group had lower $HbA_{1C}$ levels, and treated patients may have managed themselves better because there was an open label design. Hence, these results were not interpretable. Further studies would be useful.

Unnaturally high, or supraphysiological, doses of IGF-I can cause side effects (reviewed in Ishii and Pu, 1999). High doses of IGF-I (60 to 220 $\mu$g/kg/day, subcutaneous) intended to prevent hyperglycemia in type II diabetic patients may cause side effects, including hypoglycemia, arthalgia, myalgia, edema, bilateral jaw tenderness, tachycardia, papilledema, orthostatic hypotension, nausea, facial pallor or flushing, hand tremors, headaches, dyspnea, and hand erythema. These untoward effects were all reversible following withdrawal of IGF treatment. IGF-I doses below 50 $\mu$g/kg/day are well tolerated.

IGF-II is not, but IGF-I is, associated with prostate cancer (Chan et al., 1998). IGF-I is also associated with breast cancer. IGF-I levels may be elevated because tumors produce IGF-I. Similarly, insulin is a mitogen and is produced by some tumors. The safety issues for IGFs and insulin have many similarities. For example, normal humans have approximately 150 ng/mL IGF-I and 400 ng/mL IGF-II in their circulation, but normal humans do not have an abnormal risk of cancer. Generally cancer risk increases with aging when IGF-I levels are reduced (Tan and Baxter, 1986). In diabetic neuropathy, patients have 50% reduced levels of IGF-I, and replacement therapy intended to normalize IGF levels is not expected to increase cancer risk. Returning IGF levels to normal in diabetic patients seems likely to be associated with normal, not excessive, risk. Interestingly, diabetic patients have been treated for decades with insulin, and the high doses of insulin at the subcutaneous injection site would cross-occupy type I IGF receptors, but this is not known to increase cancer risk.

Growth hormone is used clinically and can elevate IGF-I levels. Cohen et al. (2000) have reviewed the risks and concluded that causality has not been established between cancer risk and IGF-I levels, and that growth hormone–treated patients in which IGF-I levels are brought up to normal levels should not have cancer risk increased above the normal population. Circulating IGF-I appears to have less effect on tissue growth than IGF-I produced within tissues. This is clear because inactivation of the IGF-I gene exclusively in liver, which results in 70% reduction of circulating IGF-I, has no effect on body weight or length in mice (Yakar et al., 1999). This result suggests that the administration of IGF to increase circulating levels is much less likely to cause cancer risk than experimental procedures in which the IGF gene is overexpressed in tissues of transgenic animals.

Nevertheless, there is the important issue of whether IGF may cause progression of existing tumors. Colon adenocarcinomas developed more rapidly when transplanted to mice with higher IGF-I levels (Wu et al., 2002). As in other forms of hormone replacement therapy, it would seem prudent that IGF-I be contraindicated in existing tumors.

### CONCLUSIONS

A decline in IGF activity is proposed to contribute to the pathogenesis of diabetic neurological complications (Ishii, 1995). This theory provides a rational basis both for the design of testable hypotheses and therapeutic treatment. The theory predicts with a high success rate that IGF treatment, intended to replace the IGF levels diminished in diabetes, can prevent or reverse diabetic neurological disturbances, particularly those neural functions normally governed by IGFs. The observation that IGF replacement therapy can prevent or reverse neurological disturbances in both the central and peripheral nervous systems in diabetes is a powerful indication that these neurological disturbances share a common etiology, namely loss of IGF activity. Heretofore, the literature on central and peripheral neurological diabetic disturbances have been separate, and the new data may help merge these fields of investigation.

The theory further predicts and finds that IGF may prevent neurological disturbances independently of ongoing hyperglycemia. Clinical trial shows that the progression of renal disease likewise can be prevented in diabetic patients by treatments that do not target glycemic control. These data together show that a large number of complex biochemical pathways involving pathogenetic processes in peripheral nerve, brain, and kidneys are *not* blocked by the consequences of hyperglycemia. Disturbances to these pathways can be prevented and/or reversed by increasing or inhibiting growth factor responses independently of ongoing hyperglycemia. This realization may help fuel the development of new therapeutic approaches to treat diabetic complications.

The pharmacokinetic and safety profiles of IGFs are satisfactory for consideration of clinical trials for IGF treatment of diabetic neuropathy. A major challenge is to find support for clinical trials. For example, there is no mechanism by which the National Institutes of Neurological Disorders and Stroke or National Institute of Diabetes, Digestive and Kidney Disorders can fund the acquisition of clinical grade IGFs for trials. The cost of drug acquisition can exceed the other costs of a clinical trial. A change in Institute policy is needed to permit basic research developed under National Institutes of Health (NIH)-funded research to be rapidly moved into the clinics. The National Cancer Institute provides a model by which manufacturing might be accomplished. It supports a center that synthesizes promising small drug candidates under manufacturing guidelines set by the Food and Drug Administration (FDA). A similar center is needed for the synthesis of promising therapeutic proteins. The successful human genome project is likely to uncover many additional promising therapeutic proteins, and the efficient testing of new therapies will be greatly aided by the availability of an NIH-supported center for the production of clinical grade therapeutic proteins.

### REFERENCES

Acerini, C. L., Patton, C. M., Savage, M. O., Kernell, A., Westphal, O., and Dunger, D. B. (1997) Randomised placebo-controlled trial of human recombinant insulin-like growth factor I plus intensive insulin therapy in adolescents with insulin-dependent diabetes mellitus. *Lancet,* **350,** 1199–1204.

American Diabetes Association. (1993) *Diabetes 1993 Vital Statistics*.

Araki, Y., Nomura, M., Tanaka, H., Yamamoto, H., Yamamoto, T., Tsukaguchi, I., and Nakamura, H. (1994) MRI of the brain in diabetes mellitus. *Neuroradiology,* **36,** 101–103.

Armstrong, C. S., Wuarin, L., and Ishii, D. N. (2000) Uptake of circulating insulin-like growth factor-I into the cerebrospinal fluid of normal and diabetic rats, and normalization of IGF-II mRNA content in diabetic rat brain. *J. Neurosci. Res.,* **59,** 649–660.

Arner, P., Sjoberg, S., Gjotterberg, M., and Skottner, A. (1989) Circulating insulin-like growth factor I in type 1 (insulin-dependent) diabetic patients with retinopathy. *Diabetologia,* **32,** 753–758.

Ballintine, E. J., Foxman, S., Gorden, P., et al. (1981) Rarity of diabetic retinopathy in patients with acromegaly. *Arch. Intern. Med.,* **141,** 1625–1627.

Beck, K., Powell-Braxton, L., Widmer, H., Valverde, J, and Hefti, F. (1995) IGFl gene disruption results in reduced brain size, CNS hypomyelination, and loss of hippocampal granule and striatal parvalbumin-containing neurons. *Neuron,* **14,** 717–730.

*Beilharz, E. J., Bassett, N. S., Sirimanne, E. S., Williams, C. E., and Gluckman, P. D. (1995) Insulin-like growth factor II is induced during wound repair following hypoxic-ischemic injury in the developing rat brain. *Mol. Brain Res.,* **29,** 81–91.

Biessels, G. J., Kamal, A., Ramakers, G. M., Urban, I. J., Spruijt, B. M., Erkelens, D. W., and Gispen, W. H. (1996) Place learning and hippocampal synaptic plasticity in streptozotocin-induced diabetic rats. *Diabetes,* **45,** 1259–1266.

Bild, D. E., Selby, J. V., Sinnock, P., Browner, W. S., Breveman, P., and Showstack, J. A. (1989) Lower-extremity amputations in people with diabetes. Epidemiology and prevention. *Diabetes Care,* **12,** 24–31.

Bitar, M. S., and Pilcher, C. W. (1998) Attenuation of IGF-1 antinociceptive action and a reduction in spinal cord gene expression of its receptor in experimental diabetes. *Pain,* **75,** 69–74.

Bitar, M. S., Pilcher, C. W. T., Khan, I., and Waldbillig, R. J. (1997) Diabetes-induced suppression of IGF-I and its receptor mRNA levels in rat superior cervical ganglia. *Diabetes Res. Clin. Pract.,* **38,** 73–80.

Bodkin, N. L., Sportsman, R., DiMarchi, R. D., and Hansen, B. C. (1991) Insulin-like growth Factor I in non-insulin-dependent diabetic monkeys: Basal plasma concentrations and metabolic effects of exogenously administered biosynthetic hormone. *Metabolism,* **40,** 1131–1137.

Bornfeldt, K. E., Arnqvist, H. J., Enberg, B., Mathews, L. S., and Norstedt, G. (1989) Regulation of insulin-like growth factor-I and growth hormone receptor gene expression by diabetes and nutritional state in rat tissues. *J. Endocrinol.,* **122,** 651–656.

Caroni, P., and Grandes, P. (1990) Nerve sprouting in innervated adult skeletal muscle induced by exposure to elevated levels of insulin-like growth factors. *J. Cell Biol.,* **110,** 1307–1317.

Carson, M. J., Behringer, R. R., Brinster, R. L., and McMorris, F. A. (1993) Insulin-like growth factor I increases brain growth and central nervous system myelination in transgenic mice. *Neuron,* **10,** 729–740.

Carsten, R. E., Whalen, L. R., and Ishii, D. N. (1989) Impairment of spinal cord conduction velocity in diabetic rats. *Diabetes,* **38,** 730–736.

Chait, A. (1993) Intensive therapy for NIDDM: A word of caution. *Clin. Diabetes,* **11,** 143–144.

Chan, J. M., Stampfer, M. J., Giovannucci, E., et al. (1998) Plasma IGF-I and prostate cancer risk: A prospective study. *Science,* **279,** 563–566.

Cheng, B., and Mattson, M. P. (1992) IGF-I and IGF-II protect cultured hippocampal and septal neurons against calcium-mediated hypoglycemic damage. *J. Neurosci.,* **12,** 1558–1566.

Clarke, B. F., Ewing, D. J., and Campbell, I. W. (1979) Diabetic autonomic neuropathy. *Diabetologia,* **17,** 195–212.

Cogesshal, C., and Root, H. F. (1940) Acromegaly and diabetic mellitus. *Endocrinology,* **26,** 1–25.

Cohen, P., Clemmons, D. R., and Rosenfeld, R. G. (2000) Does the GH-IGF axis play a role in cancer pathogenesis? *Growth Horm. IGF Res.,* **10,** 297–305.

Cornblath, D. R., Dellon, A. L., and MacKinnon, S. E. (1989) Spontaneous diabetes mellitus in a rhesus monkey: neurophysiological studies. *Muscle Nerve,* **12,** 233–235.

Craner, M. J., Klein, J. P., Black, J. A., and Waxman, S. G. (2002) Preferential expression of IGF-I in small DRG neurons and down-regulation following injury. *Neuroreport,* **13,** 1649–1652.

Crosby, S. R., Tsigos, C., Anderton, C. D., Gordon, C., Young, R. J., and White, A. (1992) Elevated plasma insulin-like growth factor binding protein-1 levels in type 1 (insulin-dependent) diabetic patients with peripheral neuropathy. *Diabetologia,* **35,** 868–872.

Crowe, M. J., Bresnahan, J. C., Shuman, S. L., Masters, J. N., and Beattie, M. S. (1997) Apoptosis and delayed degeneration after spinal cord injury in rats and monkeys. *Nat. Med.,* **1,** 73–76.

Dejgaard, A., Gade, A., Larsson, H., Balle, V., Parving, A., and Parving, H. H. (1991) Evidence for diabetic encephalopathy. *Diabetic. Med.,* **8,** 162–167.

de Pablo, F., and de la Rosa, E. J. (1995) The developing CNS: A scenario for the action of proinsulin, insulin and insulin-like growth factors. *Trends Neurol. Sci.,* **18,** 143–150.

D'Ercole, A. J., Ye, P., Calikoglu, A. S., and Gutierrez-Ospina, G. (1996) The role of insulin-like growth factors in the central nervous system. *Mol. Neurobiol.,* **13,** 227–255.

D'Mello, S. R., Galli, C., Ciotti, T., and Calissano, P. (1993) Induction of apoptosis in cerebellar granule neurons by low potassium: inhibition of death by insulin-like growth factor I and cAMP. *Proc. natl. Acad. Sci. U. S. A.,* **90,** 10989–10993.

Diabetes Control and Complications Trial Research Group. (1993) The effect of intensive treatment of diabetes on the development and progression of long-term complications in insulin-dependent diabetes mellitus. *N. Engl. J. Med.,* **329,** 977–986.

Dore, S., Kar, S., and Quirion, R. (1997) Insulin-like growth factor I protects and rescues hippocampal neurons against $\beta$-amyloid and human amylin-induced toxicity. *Proc. Natl. Acad. Sci. U. S. A.,* **94,** 4772–4777.

Dyck, P. J., Kratz, K. M., Karnes, J. L., et al. (1993) The prevalence by staged severity of various types of diabetic neuropathy, retinopathy, and nephropathy in a population-based cohort: The Rochester Diabetic Neuropathy Study. *Neurology,* **43,** 817–824.

Dyck, P. J., Thomas, P. K., Asbury, A. K., Winegrad, A. I., and Porte, D., Jr. (1987) *Diabetic Neuropathy*. Philadelphia, WB Saunders.

Ekman, B., Nystrom, F., and Arnqvist, H. J. (2000) Circulating IGF-I concentrations are low and not correlated to glycaemic control in adults with type 1 diabetes. *Eur. J. Endocrinol.,* **143,** 505–510.

Ekstrom, P. A. R., and Tomlinson, D. R. (1989) Impaired nerve regeneration in streptozotocin-diabetic rats. Effects of treatment with an aldose reductase inhibitor. *J. Neurol. Sci.,* **93,** 231–237.

Fagin, J. A., Roberts, C. T. Jr., LeRoith, D., and Brown, A. T. (1989) Coordinate decrease of tissue insulin-like growth factor I post-transcriptional alternative mRNA transcripts in diabetes mellitus. *Diabetes,* **38,** 428–434.

Fernandez, A. M., Gonzalez de la Vega, A., and Torres-Aleman, I. (1998) Insulin-like growth factor restores motor coordination in a rat model of cerebellar ataxia. *Proc. Natl. Acad. Sci. U. S. A.,* **95,** 1253–1258.

Gao, W.-Q., Shinsky, N., Ingle, G., Beck, K., Elias, K. A., and Powell-Braxton, L. (1999) IGF-I deficient mice show reduced peripheral nerve conduction velocities and decreased axonal diameters and respond to exogenous IGF-I treatment. *J. Neurobiol.,* **39,** 142–152.

Gehrs, K. M. (2001) Efficacy of octreotide in the therapy of severe nonproliferative and early proliferative diabetic retinopathy: A randomized conrolled study. *Diabetes Care,* **24,** 182.

Gerhardinger, C., McClure, K. D., Romeo, G., Podesta, F., and Lorenzi, M. (2001) IGF-I mRNA and signaling in the diabetic retina. *Diabetes,* **50,** 175–183.

Glazner, G. W., and Ishii, D. N. (1995) Insulinlike growth factor gene expression in rat muscle during reinnervation. *Muscle Nerve,* **18,** 1433–1442.

Glazner, G. W., Lupien, S., Miller, J. A., and Ishii, D. N. (1993) Insulin-like growth factor-II increases the rate of sciatic nerve regeneration in rats. *Neuroscience,* **54,** 791–797.

Glazner, G. W., Morrison, A. E., and Ishii, D. N. (1994) Elevated insulin-like growth factor (IGF) gene expression in sciatic nerves during IGF-supported nerve regeneration. *Mol. Brain Res,* **25,** 265–272.

Gluckman, P., Klempt, N., Guan, J., Mallard, C., et al. (1992) A role for IGF-1 in the rescue of CNS neurons following hypoxic-ischemic injury. *Biochem. Biophys. Res. Commun.* **182,** 593–599.

Goya, L., de la Puente, A., Ramos, S., Martin, M. A., Escriva, F., Alvarez C., and Pascual-Leone, A. M. (2001) Regulation of IGF-I and -II by insulin in primary cultures of fetal rat hepatocytes. *Endocrinology,* **142,** 5089–5096.

Grant, M. B., Mames, R. N., Fitzger, C., Hazariwala, K. M., Cooper-DeHoff R, Caballer S., and Estes K. S. (2000) The efficacy of octreotide in the therapy of severe nonproliferative and early proliferative diabetic retinopathy. *Diabetes Care,* **23,** 504–509.

Guo, H., Yang, Y., Geng, Z., Zhu, L., Yuan, S., Zhao, Y., Gao, Y., and Fu, H. (1999) The changes of insulin-like growth factor-1 in diabetic patients with neuropathy. *Chin. Med. J.,* **112,** 76–79.

Hall, K., and Sara, V. R. (1984) Somatomedin (IGF) levels in childhood, adolescence and adult life. *Clin. Endocrinol. Metab,* **13,** 91–112.

Haruta, T., Kobayashi, M., Takata, Y., Ishibashi, O., and Shigeta, Y. (1989) Insulin-like growth factor I receptors on erythrocytes in NIDDM. *Diabetes Res. Clin. Pract.,* **6,** 95–101.

Hatton, J., Rapp, R. P., Kudsk, K. A., et al. (1997) Intravenous insulin-like growth factor-I (IGF-I) in moderate-to-severe head injury: a phase II safety and efficacy trial. *J. Neurosurg.* **86,** 779–786.

Henricsson, M., Berntorp, K., Berntorp, E., Fernlund, P., and Sundkvist, G. (1999) Progression of retinopathy after improved metabolic control in type 2 diabetic patients: Relation to IGF-1 and hemostatic variables. *Diabetes Care,* **22,** 1944–1949.

Hoeldtke, R. D., Boden, G., Shuman, C. R., and Owen, O. E. (1982) Reduced epinephrine secretion and hypoglycemia unawareness in diabetic autonomic neuropathy. *Ann. Intern. Med,* **96,** 459–462.

Hoffman, J. (1964) Peripheral neuropathy in children with diabetes mellitus. *Acta Neurol. Scand.,* **40(Suppl. 8)**, 1–23.

Hopkins, K. (1997) Show me where it hurts: Tracing the pathways of pain. *J. N. I. H. Res.,* **9,** 37–43.

Iarovici, D. (1993) Intensive therapy is sweet news for diabetics. *J. N. I. H. Res.,* **5,** 37–39.

Ishii, D. N. (1989) Relationship of insulinlike growth factor-II gene expression in muscle to synaptogenesis. *Proc. Natl. Acad. Sci. U. S. A.,* **86,** 2898–2902.

Ishii, D. N. (1992) Neurobiology of insulin and insulin-like growth factors. In: *Neurotrophic Factors*, Edited by Loughlin, S. E., and Fallon, J. H., pp. 415–442. New York, Academic Press.

Ishii, D. N . (1993) Insulin and related neurotrophic factors in diabetic neuropathy. *Diabetic Med.,* **10(Suppl. 2)**, 14S–15S.

Ishii, D. N. (1995) Implication of insulin-like growth factors in the pathogenesis of diabetic neuropathy. *Brain Res. Rev.,* **20,** 47–67.

Ishii, D. N., Glazner, G. W., and Pu, S.-F. (1994) Role of insulin-like growth factors in peripheral nerve regeneration. *Pharmacol. Ther.,* **62,** 125–144.

Ishii, D. N., Glazner, G. W., Wang, C., and Fernyhough, P. (1989) Neurotrophic effects and mechanism of insulin, insulin-like growth factors, and nerve growth factor in spinal cord and peripheral neurons. In: *Molecular and Cellular Biology of Insulin-Like Growth Factors and Their Receptors: Implications for the CNS*, Edited by LeRoith, D., and Raizada, M. K., pp. 403–425. New York, Plenum Publishing.

Ishii, D. N., Glazner, G. W., and Whalen, L. R. (1993) Regulation of peripheral nerve regeneration by insulin-like growth factors. *Ann. N. Y. Acad. Sci.,* **692,** 172–183.

Ishii, D. N., Guertin, D., and Whalen, L. R. (1994) Reduced insulin-like growth factor-I mRNA content in liver, adrenal glands and spinal cord of diabetic rats. *Diabetologia,* **37,** 1073–1081.

Ishii, D. N., and Lupien, S. (1995) Insulin-like growth factors protect against diabetic neuropathy: Effects on sensory nerve regeneration in rats. *J. Neurosci. Res.,* **40,** 138–144.

Ishii, D. N., and Marsh, D. J. (1993) On the therapeutic potential for insulin-like growth factor use in motor neuron disease. *Exp. Neurol.,* **124,** 96–99.

Ishii, D. N., and Mill, J. M. (1987) Molecular mechanism of neurite formation stimulated by insulinlike factors and nerve growth factor. *Curr. Topics Membranes Transport,* **31,** 31–78.

Ishii, D. N., and Pu, S.-F. (1999) Neuropharmacology of insulin-like growth factors. *Handbook Exp. Pharmacol.,* **134,** 119–146.

Ishii, D. N., Recio-Pinto, E., Spinelli, W., Mill, J. F., and Sonnenfeld, K. H. (1985) Neurite formation modulated by nerve growth factor, insulin, and tumor promoter receptors. *Int. J. Neurosci.,* **26,** 109–127.

Ishii, D. N., Wang, C., and Li, Y. (1991) Second messengers mediating gene expression essential to neurite formation directed by insulin and insulin-like growth factors. *Adv. Exp. Med. Biol.,* **293,** 361–378.

Kanje, M., Skottner, A., Sjoberg, J., and Lundborg, G. (1989) Insulin-like growth factor I (IGF-I) stimulates regeneration of the rat sciatic nerve. *Brain Res.,* **486,** 396–398.

Kitraki, E., Bozas, E., Phillippidis, H., and Stylianopoulou, F. (1993) Aging-related changes in IGF-II and C-Fos gene expression in the rat brain. *Int. J. Dev. Neurosci.,* **11,** 1–9.

Knusel, B., Michel, P. P., Schwaber, J. S., and Hefti, F. (1990) Selective and nonselective stimulation of central cholinergic and dopaminergic development in vitro by NGF, bFGF, EGF, insulin and the IGFs I and II. *J. Neurosci.,* **10,** 558–570.

Korolkiewicz, R. P., Jujita, A., Seto, K., Suzuki, K., and Takeuchi, K. (2000) Polaprezinc exerts a salutary effect on impaired healing of acute gastric lesions in diabetic rats. *Digest. Dis. Sci.,* **45,** 1200–1209.

Kulik, G., Klippel, A., and Weber, M. J. (1997) Antiapoptotic signalling by the insulin-like growth factor I receptor, phosphatidylinositol 3-kinase, and akt. *Mol. Cell Biol.,* **17,** 1595–1606.

Kummer, A., Ishii, D. N., Pulford, B. E., and Seigel, G. M. (2003) Des(1–3)IGF-1 treatment normalizes type 1 IGF receptor, and phospho-Akt (Thr 308) immunoreactivity in pre-degenerative retina of diabetic rats. *Int. J. Exp. Diabetes Res.* (in press).

Kuzuya, H., Matsuura, N., Sakamoto, M., Makino, H, et al. (1993) Trial of insulinlike growth factor-I therapy for patients with extreme insulin resistance syndromes. *Diabetes,* **42,** 696–705.

Lewis, E. J., Hunsicker, L. G., Bain, R. P., et al. (1993) The effect of angiotensin-converting-enzyme inhibition on diabetic nephropathy. *N. Engl. J. Med.,* **329,** 1456–1462.

Litzelman, D. K., Slemenda, C. W., Langefeld, C. D., Hays, L. M., Welch, M. A., Bild, D. E., Ford, E. S., and Vinicor F. (1993) Reduction of lower extremity clinical abnormalities in patients with non-insulin-dependent diabetes mellitus. *Ann. Intern. Med,* **119,** 36–41.

Longo, F. M., Powell, H. C., Lebeau, J., Gerrero, M. R., Heckman, H., and Myer, R. R. (1986) Delayed nerve regeneration in streptzotocin diabetic rats. *Muscle Nerve,* **9,** 385–393.

Lowe, W. L., Florkiewicz, R. Z., Yorek, M. A., Spanheimer, R. G., and Albrecht, B. N. (1995) Regulation of growth factor mRNA levels in the eyes of diabetic rats. *Metabolism,* **44,** 1038–1045.

Lupien, S. B., Bluhm, E. J., and Ishii, D. N. (2002) Subcutaneous IGF-I administration normalizes water maze performance in diabetic rats. *Soc. Neurosci. Abstract,* 450.15.

Mann, D. M., Yates, P. O., and Hawkes, J. (1983) The pathology of the human locus ceruleus. *Clin. Neuropathol.,* **2,** 1–7.

Mathews, L. S., Hammer, R. E., Behringer, R. R., D'Ercole, A. J., Bell, G. I., Brinster, R. L., and Palmiter, R. D. (1988) Growth enhancement of transgenic mice expressing human insulin-like growth factor I. *Endocrinology,* **123,** 2827–2833.

Migdalis, I. N., Kalogeropoulou, K., Kalantzis, L., Nounopoulos, C., Bouloukos, A., and Samartzis, M. (1995) Insulin-like growth factor-I and IGF-I receptors in diabetic patients with neuropathy. *Diabetic Med.,* **12,** 823–827.

Mill, J. F., Chao, M. V., and Ishii, D. N. (1985) Insulin, insulinlike growth factor-II and nerve growth factor effects on tubulin mRNA levels and neurite formation. *Proc. Natl. Acad. Sci. U. S. A.,* **82,** 7126–7130.

Moller, D. E. (1995) Transgenic approaches to the pathogenesis of NIDDM. *Diabetes,* **43,** 1394–1401.

Near, S. L., Whalen, L. R., Miller, J. A., and Ishii, D. N. (1992) Insulin-like growth factor II stimulates motor nerve regeneration in rats. *Proc. Natl. Acad. Sci. U. S. A.,* **89,** 11716–11720.

Neff, N. T., Prevette, D., Houenou, L. J., Lewis, M. E., Glicksman, M. A., Yin, Q. W., and Oppenheim, R. W. (1993) Insulin-like growth factors: Putative muscle-derived trophic agents that promote motoneuron survival. *J. Neurobiol.,* **24,** 1578–1588.

Noguchi, T., Sekiguchi, M., Sugisaki, T., Tsukada, Y., and Shimai, K. (1983) Faulty development of cortical neurons in the Snell dwarf cerebrum. *Dev. Brain Res.,* **10,** 125–138.

Ott, A., Stolk, R. P., van Harskamp, F., Pols, H. A. P., Hofman, A., and Breteler, M. M. B. (1999) Diabetes mellitus and the risk of dementia. *Neurology,* **53,** 1937–1942.

Parrizas, M., and LeRoith, D. (1997) Insulin-like growth factor-1 inhibition of apoptosis is associated with increased expression of the bcl-X gene product. *Endocrinology,* **138,** 1355–1358.

Parving, H. H., Lehnert, H., Brochner-Mortensen, J., et al. (2001) The effect of irbesartan on the development of diabetic nephropathy in patients with type 2 diabetes. *N. Engl. J. Med.,* **345,** 870–878.

Perlmuter, L. C., Hakami, M. K., Hodgson-Harrington, C., Ginsberg, J., Katz, J., Singer, D. E., and Nathan, D. M. (1984) Decreased cognitive function in aging non-insulin-dependent diabetic patients. *Am. J. Med.,* **77,** 1043–1048.

Phillips, L. S., Goldstein, S., and Pao, C. I. (1991) Nutrition and somatomedin. XXVI. Molecular regulation of IGF-I by insulin in cultured rat hepatocytes. *Diabetes,* **40,** 1525–1530.

Pirart, J. (1978) Diabetes mellitus and its degenerative complications: a prospective study of 4,400 patients observed. *Diabetes Care,* **1,** 168–188.

Pu, S.-F., Zhuang, H.-X., and Ishii, D. N. (1995) Differential spatiotemporal expression of the insulin-like growth factor genes in regenerating sciatic nerve. *Mol. Brain Res,* **34,** 18–28.

Pu, S.-F., Zhuang, H.-X., Marsh, D. J., and Ishii, D. N. (1999a) Insulin-like growth factor-II increases and IGF is required for postnatal rat spinal motoneuron survival following sciatic nerve axotomy. *J. Neurosci. Res.,* **55,** 9–16.

Pu, S.-F., Zhuang, H.-X., Marsh, D. J., and Ishii, D. N. (1999b) Time-dependent alteration of insulin-like growth factor gene expression during nerve regeneration in regions of muscle enriched with neuromuscular junctions. *Mol. Brain Res.,* **63,** 207–216.

Pulford, B. E., and Ishii, D. N. (2001) Uptake of circulating insulin-like growth factors (IGFs) into cerebrospinal fluid appears to be independent of the IGF receptors as well as IGF-binding proteins. *Endocrinology,* **142,** 213–220.

Pulford, B. E., Whalen, L. R., and Ishii, D. N. (1999) Peripherally administered insulin-like growth factor-I preserves hindlimb reflex and spinal cord noradrenergic circuitry following a central nervous system lesion in rats. *Exp. Neurol.,* **159,** 114–123.

Rabinovsky, E. D., Kattash, M. M., Shenaq, S. M., DeMayo, F., and Schwartz, R. (1996) The role of IGF-I in peripheral nerve regeneration: Studies in IGF-I transgenic mice. *Soc. Neurosci. Abstract,* **22:** 1960.

Recio-Pinto, E., and Ishii, D. N. (1984) Effects of insulin, insulin-like growth factor-II and nerve growth factor on neurite outgrowth in cultured human neuroblastoma cells. *Brain Res,* **302,** 323–334.

Recio-Pinto, E., and Ishii, D. N. (1988) Insulin and related growth factors: Effects on the nervous system and mechanism for neurite growth and regeneration. *Neurochem. Int.,* **12,** 397–414.

Recio-Pinto, E., Lang, F. F., and Ishii, D. N. (1984) Insulin and insulin-like growth factor II permit nerve growth factor binding and the neurite formation response in cultured human neuroblastoma cells. *Proc. Natl. Acad. Sci. U. S. A.,* **81,** 2562–2566.

Recio-Pinto, E., Rechler, M. M., and Ishii, D. N. (1986) Effects of insulin, insulinlike growth factor-II, and nerve growth factor on neurite formation and survival in cultured sympathetic and sensory neurons. *J. Neurosci.,* **6,** 1211–1219.

Reinhardt, R. R., and Bondy, C. A. (1994) Insulin-like growth factors cross the blood-brain barrier. *Endocrinology,* **135,** 1753–1761.

Rieu, M., and Binoux, M. (1985) Serum levels of insulin-like growth factor (IGF) and IGF binding protein in insulin-dependent diabetics during an episode of severe metabolic decompensation and the recovery phase. *J. Clin. Endocrinol. Metab.,* **60,** 781–785.

Ryan, C. M., Williams, T. M., Finegold, D. N., and Orchard, T. J. (1993) Cognitive dysfunction in adults with type 1 (insulin-dependent) diabetes mellitus of long duration: Effects of recurrent hypoglycaemia and other chronic complications. *Diabetologia,* **36,** 329–334.

Saatman, K. E., Contreras, P. C., Smith, D. H., et al. (1997) Insulin-like growth factor-I (IGF-I) improves both neurological motor and cognitive outcome following experimental brain injury. *Exp. Neurol.,* **147,** 418–427.

Santiago, J. V., White, N. H., Skor, D. A., Levandoski, L. A., Bier, D. M., and Cryer, P. E. (1984) Defective glucose counterregulation limits intensive therapy of diabetes mellitus. *Am. J. Physiol.,* **247,** E215–E220.

Schmidt, R. E., Dorsey, D. A., Beaudet, L. N., Plurad, S. B., Parvin, C. A., and Miller, M. S. (1999) Insulin-like growth factor I reverses experimental diabetic autonomic neuropathy. *Am. J. Pathol.,* **155,** 1651–1660.

Schmidt, R. E., Plurad, S. B., Parvin, C. A., and Roth, K. A. (1993) The effect of diabetes and aging on human sympathetic autonomic ganglia. *Am. J. Pathol.,* **143,** 143–153.

Sherrard, R. M. (1997) Insulin-like growth factor I induces climbing fibre re-innervation of the rat cerebellum. *Neuroreport,* **8,** 3225–3228.

Shimatsu, A., and Rotwein, P. S. (1987) Mosaic evolution of the insulin-like growth factors. Organization, sequence and expression of the rat insulin-like growth factor I gene. *J. Biol. Chem.,* **262,** 7894–7900.

Sima, A. A. F., Thomas, P. K., Ishii, D. N., and Vinik, A. (1997) Diabetic neuropathies. *Diabetologia,* **40,** B74–B77.

Simo, R., Lecube, A., Segura, R. M., Arumi, J. G., and Hernandez, C. (2002) Free insulin growth factor-I and vascular endothelial growth factor in the vitreous fluid of patients with proliferative diabetic retinopathy. *Am. J. Ophhalmol.,* **134,** 376–382.

Soares, M. B., Ishii, D. N., and Efstratiadis, A. (1985) Developmental and tissue-specific expression of a family of transcripts related to rat insulin-like growth factor II mRNA. *Nucleic Acids Res.,* **13,** 1119–1134.

Soares, M. B., Turken, A., Ishii, D. N., Mills, L., Episkopou, V., Cotter, S., Zeitlin, S., and Efstratiadis, A. (1986) Rat insulin-like growth factor II gene: A single gene with two promoters expressing a multitranscript family. *J. Mol. Biol.,* **192,** 737–752.

Sonken, P. H., Jones, D. R., and Jones, R. H. (1993) Growth hormone and diabetes mellitus. *Horm. Res.,* **40,** 68–79.

Spranger, J., Buhnen, J., Jansen, V., Krieg, M., Meyer-Schwickerath, R., Blum, W. F., Schatz, H., and Pfeiffer, A. F. T. (2000) Systemic levels contribute significantly to increased intraocular IGF-I, IGF-II and IGF-BP3 in proliferative diabetic retinopathy. *Horm. Metab. Res.,* **32,** 196–200.

Tan, K., and Baxter, R. C. (1986) Serum insulin-like growth factor I levels in adult diabetic patients: The effect of age. *J. Clin. Endocrinol. Metab.,* **63,** 651–655.

Teutsch, S. M., Herman, W. H., Dwyer, D. M., and Lane, J. M. (1984) Mortality among diabetic patients using continuous subcutaneous insulin-infusion pumps. *N. Engl. J. Med.,* **310,** 361–368.

Thomas, P. K., and Tomlinson, D. R. (1993) Diabetic and hypoglycemic neuropathy. In: *Peripheral Neuropathy*, 3rd ed., Edited by Dyck, P. J., Thomas, P. K., Griffin, J. W., Low, P. A., and Poduslo, J. F., pp. 1219–1250. Philadelphia, W. B. Saunders.

Torres-Aleman, I., Barrios, V., Lledo, A., and Berciano, J. (1996) The insulin-like growth factor I system in cerebellar degeneration. *Ann. Neurol.,* **39,** 335–342.

Torres-Aleman, I., Pons, S., and Santos-Benito, F. F. (1992) Survival of Purkinje cells in cerebellar cultures is increased by insulin-like growth factor I. *Eur. J. Neurosci.,* **4,** 864–869.

Tun, P. A., Nathan, D. M., and Perlmuter, L. C. (1990) Cognitive and affective disorders in elderly diabetics. *Clin. Geriatr. Med.,* **6,** 731–746.

UK Prospective Diabetes Study Group. (1998) Intensive blood-glucose control with sulphonylureas or insulin compared with conventional treatment and risk of complications in patients with type 2 diabetes. *Lancet,* **352,** 837–853.

Vinik, A. I., Newlon, P., Milicevic, Z., McNitt, P., and Stansberry, K. B. (1996) Diabetic neuropathies: An overview of clinical aspects. In: *Diabetes Mellitus a Fundamental and Clinical Text*, Edited by LeRoith, D., Taylor, S. I., and Olefsky, J. M., pp. 737–751. Philadelphia, Lippincott-Raven.

Wang, C., Li, Y., Wible, B., Angelides, K., and Ishii, D. N. (1992) Neurofilament mRNA and tubulin mRNA content in human neuroblastoma SH-SY5Y cells during neurite outgrowth directed by insulin and insulin-like growth factors. *Mol. Brain Res.,* **13,** 289–300.

Wang, Q., Dills, D. G., Klein, R., Klein, B. E. K., and Moss, S. E. (1995) Does insulin-like growth factor 1 predict incidence and progression of retinopathy? *Diabetes*, **44,** 161–164.

Wojnar, M. M., Fan, J., Frost, R. A., Gelato, M. C., and Lang, C. H. (1995) Alterations in the insulin-like growth factor system in trauma patients. *Am. J. Physiol.,* **268,** R970–R977.

Wuarin, L., Guertin, D. M., and Ishii, D. N. (1994) Early reduction in insulin-like growth factor gene expression in diabetic nerve. *Exp. Neurol.,* **130,** 104–114.

Wuarin, L., Namdev, R., Burns, J. G., Fei, Z.-J., and Ishii, D. N. (1996) Brain insulin-like growth factor-II mRNA content is reduced in insulin-dependent and non-insulin-dependent diabetes mellitus. *J. Neurochem.,* **67,** 742–751.

Yakar, S., Liu, J. L., Stannard, B., Butler, A., Accili, D., Sauer, B., and LeRoith, D. (1999) Normal growth and development in the absence of hepatic insulin-like growth factor I. *Proc. Natl. Acad. Sci. U. S. A.,* **96,** 7324–7329.

Yao, D. L., Liu, X., Hudson, L. D., and Webster, H. D. (1995) Insulin-like growth factor I treatment reduces demyelination and up-regulates gene expression of myelin-related proteins in experimental autoimmune encephalomyelitis. *Proc. Natl. Acad. Sci. U. S. A.,* **92,** 6190–6195.

Zhang, W., Ghetti, B., and Lee, W. H. (1997) Decreased IGF-I gene expression during the apoptosis of Purkinje cells in pcd mice. *Dev. Brain Res.,* **98,** 164–176.

Zhuang, H.-X., Snyder, C. K., Pu, S.-F., and Ishii, D. N. (1996) Insulin-like growth factors reverse or arrest diabetic neuropathy: Effects on hyperalgesia and impaired nerve regeneration in rats. *Exp. Neurol.,* **140,** 198–205.

Zhuang, H.-X., Wuarin, L., Fei, Z.-J., and Ishii, D. N. (1997) Insulin-like growth factor (IGF) gene expression is reduced in neural tissues and liver from rats with non-insulin-dependent diabetes mellitus, and IGF treatment ameliorates diabetic neuropathy. *J. Pharmacol. Exp. Ther.,* **283,** 366–374.

